# Esophagectomy versus definitive chemoradiotherapy for patients with clinical stage N0 and pathological stage T1b esophageal squamous cell carcinoma after endoscopic submucosal dissection: study protocol for a multicenter randomized controlled trial (Ad-ESD Trial)

**DOI:** 10.1186/s13063-020-04461-5

**Published:** 2020-07-01

**Authors:** Yang Yang, Yuchen Su, Xiaobin Zhang, Jun Liu, Hong Zhang, Bin Li, Rong Hua, Lijie Tan, Hezhong Chen, Zhigang Li

**Affiliations:** 1 Department of Thoracic Surgery, Shanghai Chest Hospital, Shanghai Jiao Tong University, No.241 West Huaihai Road, Shanghai, 200030 China; 2 Department of Endoscopy, Shanghai Chest Hospital, Shanghai Jiao Tong University, No.241 West Huaihai Road, Shanghai, 200030 China; 3 Department of Radiation Oncology, Shanghai Chest Hospital, Shanghai Jiao Tong University, No.241 West Huaihai Road, Shanghai, 200030 China; 4 Department of Thoracic Surgery, Zhongshan Hospital, Fudan University, No. 180 Fenglin Road, Shanghai, 200032 China; 5 Department of Thoracic Surgery, Changhai Hospital, Naval Military Medical University, No. 168 Changhai Road, Shanghai, 200433 China

**Keywords:** Esophageal cancer, Submucosal lesion, Endoscopic submucosal dissection, Definitive chemoradiotherapy, Esophagectomy, Randomized controlled trial

## Abstract

**Background:**

Esophagectomy is still advised as an additional treatment for pathological T1b (pT1b) esophageal squamous cell carcinoma (ESCC) following attempted endoscopic resection (ER). ER followed with definitive chemoradiotherapy (dCRT) has shown increased quality of life as well as comparable oncological outcomes to esophagectomy. However, there is no well-designed phase III trial to compare the two treatments for patients with pT1b ESCC.

**Methods:**

One hundred seventy-six patients with clinical stage N0 (cN0) and pT1b ESCC will be recruited at three centers and randomly assigned to the esophagectomy group or the dCRT group. The clinical lymph node status will be measured by image examination, including computer tomography and positron emission tomography–computed tomography. The pathological tumor status will be diagnosed after endoscopic submucosal dissection (ESD). All patients will be followed up for 60 months after randomization. The primary endpoint is 5-year overall survival. The secondary endpoints are quality of life, related adverse events, 3-year overall survival, and relapse-free survival rates.

**Discussion:**

To the best of our knowledge, this is the first phase III randomized controlled trial to compare esophagectomy and dCRT for patients with cN0-pT1b ESCC after ESD. Based on the results of this study, we will show whether dCRT will benefit patients more than esophagectomy, which will contribute more high-quality evidence to the primary salvage treatment for these patients.

**Trial registration:**

ClinicalTrials.gov, NCT04135664. Registered on Aug. 10, 2019.

## Administrative information

**Table:** 

Title {1}	Esophagectomy versus Definitive Chemoradiotherapy for Patients with Clinical Stage N0 and Pathological Stage T1b Esophageal Squamous Cell Carcinoma After Endoscopic Submucosal Dissection: Study Protocol for A Multicenter Randomized Controlled Trial (Ad-ESD Trial)
Trial registration {2a and 2b}	ClinicalTrials.gov, NCT04135664. Registered on Aug. 10, 2019.
Protocol version {3}	Oct. 25, 2019. Ad-ESD protocol version 2.0.
Funding {4}	This study is funded by the Gaofeng Clinical Medicine Grant Support of Shanghai Municipal Education Commission.
Author details {5a}	Yang Yang, Department of Thoracic Surgery, Shanghai Chest Hospital, Shanghai Jiao Tong University, Shanghai, China. E-mail: yangyang321879@163.com Yuchen Su, Department of Endoscopy, Shanghai Chest Hospital, Shanghai Jiao Tong University, Shanghai, China. E-mail: inalcohol@126.com Xiaobin Zhang, Department of Thoracic Surgery, Shanghai Chest Hospital, Shanghai Jiao Tong University, Shanghai, China. E-mail: zxb5212@163.com Jun Liu, Department of Radiation Oncology, Shanghai Chest Hospital, Shanghai Jiao Tong University, Shanghai, China. E-mail: drjunliu@qq.com Hong Zhang, Department of Endoscopy, Shanghai Chest Hospital, Shanghai Jiao Tong University, Shanghai, China. E-mail: 79757393@qq.com Bin Li, Department of Thoracic Surgery, Shanghai Chest Hospital, Shanghai Jiao Tong University, Shanghai, China. E-mail: drbinlee@126.com Rong Hua, Department of Thoracic Surgery, Shanghai Chest Hospital, Shanghai Jiao Tong University, Shanghai, China. E-mail: askyou999@126.com Lijie Tan, Department of Thoracic Surgery, Zhongshan Hospital, Fudan University, Shanghai, China. E-mail: tan.lijie@zs-hospital.sh.cn Hezhong Chen, Department of Thoracic Surgery, Changhai Hospital, Naval Military Medical University, Shanghai, China. E-mail: rchenhz@126.com Zhigang Li, Department of Thoracic Surgery, Shanghai Chest Hospital, Shanghai Jiao Tong University, Shanghai, China. E-mail: zhigang_li_sch@163.com YY, YS, and XZ contributed equally to this study protocol.
Name and contact information for the trial sponsor {5b}	Zhigang Li, MD, PhD. Department of Thoracic Surgery, Shanghai Chest Hospital, Shanghai Jiao Tong University, No. 241, West Huaihai Rd., Shanghai, 200,030, China. Tel: + 86 2,122,200,000–2601. E-mail: zhigang_li_sch@163.com
Role of sponsor {5c}	ZL carried out the trial design and financial supervision.

## Introduction

### Background and rationale {6a}

Esophageal cancer is the seventh most common malignant tumor and ranks sixth in tumor-related mortality worldwide [1]. In terms of the histological subtypes, adenocarcinoma is frequently observed in Europe and the United States whereas squamous cell carcinoma is the predominant form in China [2].

Tumor invasion of the submucosa (T1b) is a watershed in the treatment of esophageal cancer from endoscopy to esophagectomy. Esophagectomy with extended lymph node dissection is recommend as the primary treatment for pT1b esophageal squamous cell carcinoma (ESCC) because of the high incidence of lymph node metastasis [3, 4]. Previous studies showed that patients with pT1b ESCC after esophagectomy had a favorable 5-year survival rate, which was about 70 ~ 73.6% [5, 6]. Although 30 ~ 50% of these patients have a risk of lymph node metastasis, more than half of them are presented with local superficial lesions [7]. In addition, esophagectomy is associated with a higher morbidity and mortality rate as well as decreased quality of life (QoL) [8]. Therefore, preserving the esophagus has always been the ultimate goal of treatment for low-risk submucosal esophageal cancer.

Previous studies have demonstrated that definitive chemoradiotherapy (dCRT) could achieve a survival rate comparable to that of esophagectomy for submucosal esophageal cancer [9]. However, local failure without distant metastasis after dCRT remains a major challenge to achieve long-term survival [10]. Endoscopic resection (ER) has been demonstrated with satisfied control for submucosal esophageal cancer without lymph node metastasis [11]. Therefore, the combination of ER and dCRT conforms to the aim of non-surgical treatment of submucosal esophageal cancer, which can maximize the removal of primary lesions and additional disposition of residual lesions or potential lymph node metastasis [12–14]. The Japan Clinical Oncology Group (JCOG) phase II trial (JCOG0508) was the only prospective clinical study conducted to evaluate the efficiency and safety of combined treatment of ER and CRT for clinical stage I ESCC [15]. The results showed that for patients with pT1b with R0 and pT1a with lymphovascular invasion (+) after ER, the 3-year overall survival (OS) was 90.7% (90% confidence interval 84.0%–94.7%) after prophylactic CRT. They concluded that the combination of ER and selective CRT should be considered as a minimally invasive treatment option for clinical stage I ESCC [16].

Considering the encouraging results of JCOG0508 and other previous studies, we hypothesized that, in comparison with esophagectomy, concurrent dCRT may achieve comparable survival results and better QoL for submucosal esophageal cancer. Therefore, we designed this randomized controlled trial (RCT) to compare the two salvage treatments for pT1b ESCC after ER.

### Objectives {7}

The aim of this trial is to compare esophagectomy versus dCRT for patients with clinical stage N0 and pathological stage T1b ESCC after endoscopic submucosal dissection (ESD).

### Trial design {8}

**Fig. 1:**
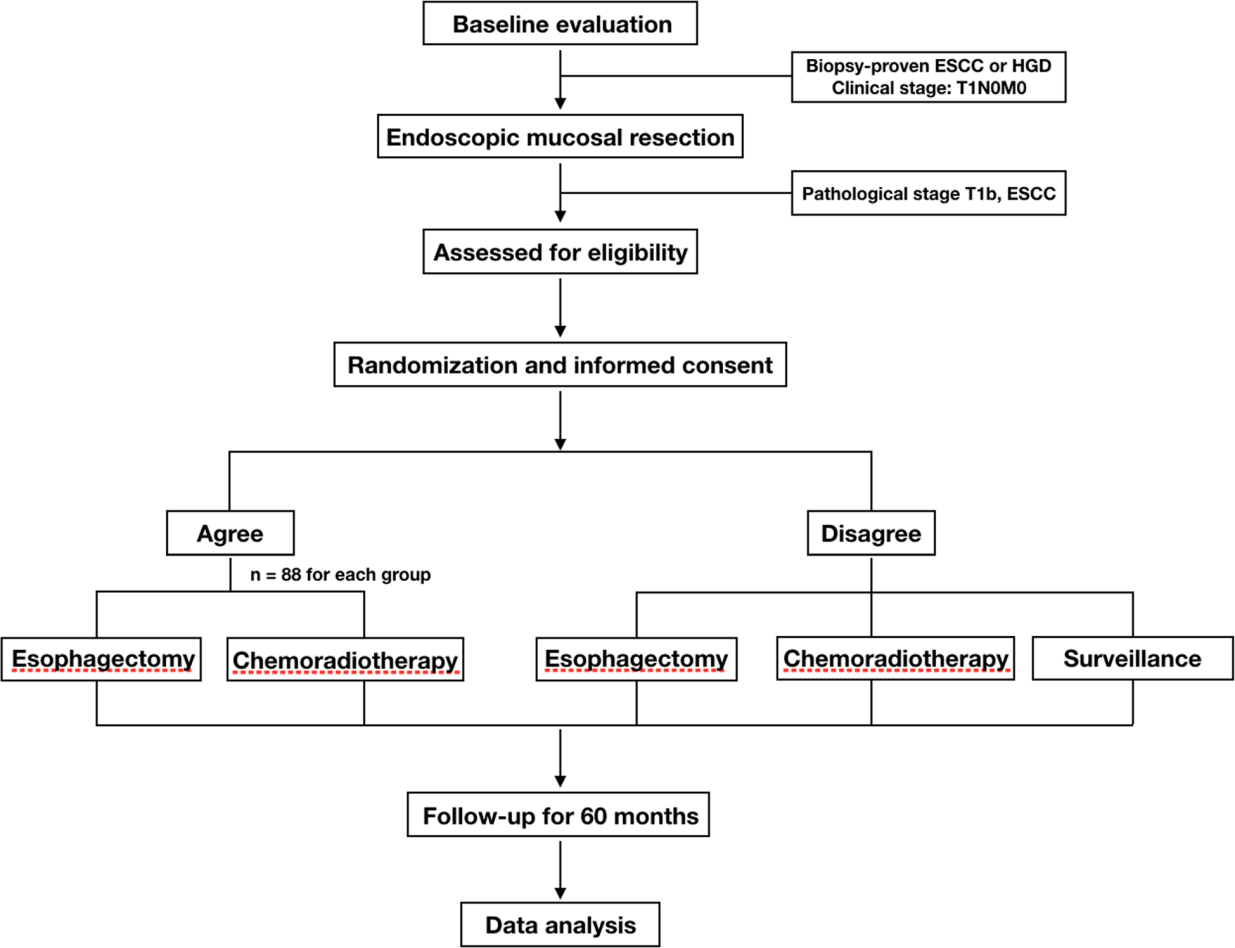
Flow chart of the trial

This study is a multicenter, randomized, open-label, phase III trial. All participants will be allocated to the two intervention groups at 1:1 ratio. The flow chart is illustrated in Fig. 1.

## Methods: Participants, interventions, and outcomes

### Study setting {9}

Patients with cN0-pT1b ESCC after ESD will receive two concurrent salvage treatments. The intervention randomly assigned with either esophagectomy or dCRT will start at about 3 weeks after ESD, followed by a 60-month follow-up period. To achieve the primary endpoint, 176 patients will be recruited from three high-volume centers (>100 cases of esophagectomies) in China (Shanghai Chest Hospital, Zhongshan Hospital, and Changhai Hospital).

### Eligibility criteria {10}

#### Inclusion criteria

1. Biopsy proven with ESCC.
2. Clinical N0 stage diagnosed by imaging examinations.
3. Pathological T1b stage confirmed by endoscopic submucosal resection.
4. Age ranges from 18 to 75 years.
5. Primary tumors are located at the intrathoracic esophagus.
6. Eastern Cooperative Oncology Group (ECOG) performance status 0–2.
7. Written informed consent.

#### Exclusion criteria

1. Prior treatment before endoscopic submucosal resection.
2. Inability to accept any treatment component.
3. Prior intervention (surgery, chemoradiation, etc.) for other primary tumor disease.
4. Positive vertical resection margin.
5. Distant metastasis.
6. Inability to understand the informed consent.

### Who will take informed consent? {26a}

The informed consent document will be obtained from the potential participants or their authorized surrogates after the screening of the inclusion and exclusion criteria. Dr. Su will explain the details of the informed consent document to the participants and their authorized surrogates. The participants will be enrolled in this trial after Dr. Su obtains written permission from participants or their authorized surrogates.

### Additional consent provisions for collection and use of participant data and biological specimens {26b}

On the consent form, participants will be asked whether they agree to the use of their data; otherwise, they could choose to withdraw from the trial. Participants will also be asked for permission for the research team to share relevant data with people from the institutions which are participating in the research or from regulatory authorities (where relevant). This trial does not involve collecting biological specimens for storage.

### Interventions

#### Explanation for the choice of comparators {6b}

The comparator of this trial is esophagectomy. The rate of lymph node metastasis in patients with pathological T1b ESCC was high (20 ~ 50%). According to the current National Comprehensive Cancer Network (NCCN) guidelines of esophageal and gastric cancer, esophagectomy is the primary treatment option for pathological T1b ESCC. The 5-year OS of patients with pT1b ESCC who underwent esophagectomy was 70 ~ 80%. So the comparator we choose for this trial is esophagectomy with lymphadenectomy.

#### Intervention description {11a}

The intervention treatments will be performed by thoracic surgeons or radiologists. Patients who meet the inclusion criteria will be recruited and randomly assigned to two treatment groups.

##### Esophagectomy

Patients will undergo an open, hybrid, or minimally invasive esophagectomy (McKeown or Ivor Lewis) with at least two-field lymphadenectomy. Selection of surgical technique will depend on patient and tumor characteristics as well as local expertise and preference [17–19]. According to the NCCN guidelines [20], the number of dissected lymph nodes should be at least 15, including the lymph nodes at the station of upper para-esophagus, right recurrent laryngeal nerve, middle para-esophagus, lower para-esophagus, left recurrent laryngeal nerve, subcarinal station, left main trachea, right main trachea, para-cardiac, left gastric artery, and lesser curve.

##### Definitive chemoradiotherapy (dCRT)

Radiotherapy will be delivered with photons (6–10 MV) in daily fractions on 5 days per week. Intensity-modulated radiation therapy based on a computed tomography (CT) simulation planning system with 5-mm-thick scan slices throughout the entire neck and thorax and upper abdomen is required.

Target volumes need to be carefully defined.

Gross tumor volume (GTV): The GTV should include the positive margin according to the pathology after ESD.

Clinical target volume (CTV): The CTV is defined as tumor bed and elective lymph node regions. For the proximal third of the esophagus, consider treatment of para-esophageal lymph nodes, bilateral supraclavicular lymph nodes, and mediastinum lymph nodes. For the middle third of the esophagus, consider treatment of para-esophageal lymph nodes. For the distal third of the esophagus, consider para-esophageal, lesser curvature, splenic nodes, and celiac axis nodal regions.

Planning target volume (PTV): The PTV includes PTV-G and PTV-C. Owing to set-up deviation and organ movement, PTV-G is defined as a further 6- to 10-mm expansion to the GTV in all directions and PTV-C is defined as a further 6- to 10-mm expansion to the CTV. The prescribed dose of PTV-G is 6020 cGy (215 cGy/d), PTV-C is 5040 cGy (180 cGy/d), both in 28 fractions.

##### Chemotherapy

The following chemotherapeutic agents were used: Cisplatin was administered at a dose of 70 mg/m^2^ by a slow drip infusion on days 1 and 29; 5-fluorouracil (5-FU) was administered at a dose of 700 mg/m^2^ per d by a continuous infusion for 24 h on days 1–4 and 29–32.

##### Criteria for discontinuing or modifying allocated interventions {11b}

Allocated interventions will not be modified as a rule, except for participants who cannot finish chemoradiation because of severe adverse events.

#### Strategies to improve adherence to interventions {11c}

Because this is a surgical RCT, there will be no further strategy to improve adherence to interventions.

#### Relevant concomitant care permitted or prohibited during the trial {11d}

In this trial, implementing psychological instruction will not require alteration to usual care pathways (including use of any medication) and these will continue for both trial arms.

#### Provisions for post-trial care {30}

All participants will be followed up over a period of 60 months or until death.

#### Outcomes {12}

##### Primary endpoint

The primary endpoint is 5-year OS in all randomly assigned patients. OS is defined as the time from the date of randomization to the day of last follow-up or death.

##### Secondary endpoints

QoL is assessed among patients by using the European Organization for Research and Treatment of Cancer Quality of Life Questionnaire C-30 (EORTC QLQ-C30) and EORTC QLQ-OES18 [21, 22]. Patients will be invited to finish the two questionnaires at the day of recruitment and the 1st, 3rd, 6th, 12th, and 24th month after randomization.

The oncological outcomes are 3-year OS and 3- and 5-year relapse-free survival (RFS). RFS is defined as the time from the date of randomization to the day of tumor recurrence, tumor progression, or patients’ death assessed up to 60 months.

### Participant timeline {13}

The time schedule of enrolment, interventions, assessments, and visits for participants is presented in the following schematic diagram.

### Sample size {14}

According to previous studies, the 5-year OS of patients with pT1b ESCC who underwent esophagectomy was 70 ~ 80% [23] whereas the rate was about 90% in patients who received ER plus chemoradiation [16]. We assumed that the 5-year OS rates were 75% in the esophagectomy group and 90% in the dCRT group. The proportion dropping out of the study is considered to be 5%. Therefore, a sample size of 88 patients in each group is required at a significance level of 5% and a power of 80%.

### Recruitment {15}

All patients with cN0-pT1b ESCC diagnosed after ESD treatment are potential candidates in this trial. The endoscopists will register all of these patients and notify the clinical research coordinator (CRC) of this trial, and the CRC will contact and tell potential candidates about this trial. After screening for the eligible criteria, the CRC will set up a meeting with potential candidates or their authorized surrogates and Dr. Su in the outpatient clinic. Dr. Su will explain the details of this trial to them and obtain their signatures.

The three institutions which are participating in this trial are high-volume medical centers with esophagectomy and ESD treatment. According to our estimation of the duration of recruitment, 80 patients will be recruited per year.

### Assignment of interventions: allocation

#### Sequence generation {16a}

The allocation sequence is according to the computer-generated random numbers.

#### Concealment mechanism {16b}

Sealed envelopes will be used in implementing the allocation sequence.

#### Implementation {16c}

The CRC of the Ad-ESD trial will generate the allocation sequence. A clinical physician will be in charge of the enrolment of participants and assign participants to each intervention. The assignment of interventions is not blinding.

#### Who will be blinded {17a}

Because of the different interventions in the two groups, the thoracic surgeons or radiologists will not be blinded to group allocation. Statistical analysts will be blind to the procedure and the results of randomization, group allocation, and intervention.

#### Procedure for unblinding if needed {17b}

This trial is an open-label RCT with only statistical analysts being blinded to the procedure and the results of randomization, group allocation, and intervention.

### Data collection and management

#### Plans for assessment and collection of outcomes {18a}

After completion of allocated treatments, patients will be followed up until death or over a period of at least 60 months. All patients will be required to send back the QoL questionnaires at the 1st, 3rd, 6th, 12th, and 24th month after randomization. The CT scan of chest and abdominal and ultrasound of the neck will be performed at 6-month intervals for the first 3 years and every year for the next 2 years after treatment. Positron emission tomography-CT will be used selectively.

#### Plans to promote participant retention and complete follow-up {18b}

A regular telephone follow-up will be performed every 3 months in the participating centers.

#### Data management {19}

Data will be entered into online encrypted database and a separate Excel form from the CRC staff. Researchers must have an authorized account to access the database.

#### Confidentiality {27}

All personal information about potential and enrolled participants will be safely maintained in order to protect confidentiality before, during, and after the trial.

#### Plans for collection, laboratory evaluation, and storage of biological specimens for genetic or molecular analysis in this trial/future use {33}

No biological specimens were collected as part of this trial.

### Statistical methods

#### Statistical methods for primary and secondary outcomes {20a}

Statistical analyses are performed using SPSS version 20.0 software (SPSS Inc., Chicago, IL, USA). The statistical analysis will be performed in accordance with both the intention-to-treat and the per-protocol principles. Survival will be estimated by Kaplan–Meier methods and analyzed using log-rank test. Continuous variables will be compared using a Student’s *t* test or Wilcoxon rank-sum test as appropriate and represented as the mean ± standard deviation or as median and range. Categorical variables will be compared using Fisher’s exact test or Wilcoxon rank-sum test as appropriate and represented as number of patients and percentage. For the analysis of QoL, parametric or non-parametric statistical methods will be used to assess the results of QLQ-C30 and QLQ-OES18 from pretreatment to 12 months, depending on the data distribution.

#### Interim analyses {21b}

An interim analysis will be performed after 80 patients have been included. The safety parameter will be analyzed by the statistical analysts. In case the stop rule is reached, the trial will be stopped immediately in all participating centers. Patients who have already been included will not undergo any further research-related tests. These patients will be scheduled for surgical resection according to the standard protocol for esophageal cancer.

Stop rule: The safety parameter is defined as peri-treatment mortality.

If the 30-day mortality after esophagectomy or the mortality in the duration of dCRT reaches 5%, then this trial will be stopped.

#### Methods for additional analyses (such as subgroup analyses) {20b}

There will be no additional analyses in this trial.

#### Methods in analysis to handle protocol non-adherence and any statistical methods to handle missing data {20c}

In case of missing data, an intention-to-treat analysis will be performed. For primary endpoint, for patients who are lost to follow-up, censored data will be the date of last follow-up. For secondary endpoints, all patients will have regular follow-up in the outpatient department and also be contacted by the CRC of this trial. Therefore, the data could be well collected as expected.

#### Plans to give access to the full protocol, participant-level data, and statistical code {31c}

All participating centers have access to the full protocol, participant-level dataset, and statistical code. The datasets analyzed during the present study are available from the corresponding author on reasonable request.

### Oversight and monitoring

#### Composition of the coordinating center and trial steering committee {5d}

Standard Protocol Items: Recommendations for Interventional Trials (SPIRIT) guidance: Composition, roles, and responsibilities of the coordinating center, steering committee, endpoint adjudication committee, data management team, and other individuals or groups overseeing the trial (if applicable). See Item 21a for the data monitoring committee (DMC).

A trial steering committee (TSC) was set up before the start of the trial. The TSC in this trial consists of the president of Shanghai Chest Hospital (Prof. Changqing Pan) and Prof. Zhigang Li, who act as a body that takes responsibility for the scientific integrity of a clinical trial, assessment of study quality and conduct, and the scientific quality of the final study report.

Dr. Su will be responsible for all aspects of local organization, including identifying potential recruits and taking consent. Dr. Li is supervising the trial and they will meet every two weeks to discuss the progress of this trial. Also, Dr. Li will hold online or face-to face meetings for investigators from all participating centers every three months to oversee conduct and progress.

#### Composition of the data monitoring committee, its role and reporting structure {21a}

A DMC has been established parallel to the finish of study protocol. In our study, the DMC consists of three experts: one thoracic surgeon (Prof. Wentao Fang), one radiologist (Prof. Xiaolong Fu), and a pathologist (Prof. Yuchen Han) from Shanghai Chest Hospital. The DMC should consider essential parts of study conduct such as protocol adherence and patient withdrawal. In most cases, safety monitoring, especially for severe adverse events, will be the major task for a DMC. Importantly, if major problems with the study conduct are observed, a DMC should consider possible recommendations to the sponsor to improve the quality of the study.

#### Adverse event reporting and harms {22}

Adverse events are defined as any undesirable experience occurring to a subject during the study, regardless of whether they are considered to be related to the treatment procedures. All adverse events reported spontaneously by the subject or observed by the investigator or his or her staff will be recorded during the period of study.

#### Frequency and plans for auditing trial conduct {23}

The project management group will audit this trial every three months, and the process will be entirely independent from investigators and the sponsor. Every three months, they will review the progress of the trial through the online electronic database system, which will be established before the start of this trial, and they will also hold meetings for all principal investigators (PIs) every six months. The trial steering group and the independent data monitoring and ethics committee meet to review conduct throughout the trial period.

#### Plans for communicating important protocol amendments to relevant parties (such as trial participants and ethics committees) {25}

If the protocol needs to be modified, the PI (Zhigang Li) will set up a discussion meeting with all investigators in all three centers. The deviations we discuss in the meeting will be recorded as a breach report form. The PI will first notify the funder of this trial of any deviation of the protocol and the reason for that. After granting the permission of the funder, the PI will notify all investigators and a copy of the revised protocol will be sent to the PI to add to the investigator site file. Meanwhile, the revised protocol will be updated in ClinicalTrials.gov, NCT04135664.

#### Dissemination plans {31a}

Authorship eligibility guidelines will follow International Committee of Medical Journal Editors guidelines. The final trial dataset will be available to the investigative team and on reasonable request.

## Discussion

In an increasing numbers of patients, superficial lesions are treated primary with ER after receiving lymph node and distant metastasis evaluation. However, the treatment choice will face a dilemma between esophagectomy and dCRT when the pathology of ER specimen reveals submucosal invasion, which is an increasingly common clinical problem [24]. In the past, the selection was based on the preferences of patient and doctor. Although previous retrospective studies have confirmed that adjuvant chemotherapy or surgery can achieve comparable long-term survival outcomes, small sample size and low proportion of pT1b patients reduced the credibility of evidence [14, 16]. The present study is the first prospective randomized controlled clinical trial to compare the salvage esophagectomy and dCRT for patients with cN0-pT1b ESCC after ESD. Our results will provide high-level evidence to establish an appropriate treatment strategy for these patients.

Superficial esophageal cancer is defined as tumor invaded into mucosal and submucosal lesion. In the retrospective studies, tumor invaded into muscular mucosal (M3) and submucosal (SM) layers are usually treated in the same way because of the high rate of lymph node metastasis [25]. However, we selected T1b as the study subject and excluded M3 patients in this trial, which is based mainly on the following considerations. First, it is difficult to be recruited since the majority of patients with mucosal myometrium after R0 resection in China will choose surveillance. Second, in this trial, we set very strict clinical evaluation criteria for lymph node status, which greatly reduced the probability of occurrence of high-risk M3, so there is no need for adjuvant therapy. Therefore, the T1b patients after ESD were selected as the study subjects.

In this trial design, we did not exclude patients with a positive lateral resection margin. Previous studies have confirmed that the tumor invasion within the submucosa can be well controlled by radiotherapy, and so for the residual lesions in the lateral resection margin after ESD, subsequent adjuvant chemoradiotherapy or surgery is sufficient to obtain local radical treatment [26]. Therefore, those patients do not need to be excluded, which was also consistent with the JCOG0508 study [16]. However, because the tumor has invaded into the muscular layer (T2), patients with a positive vertical resection margin will not be recruited.

For superficial lesions, the lymph node metastasis is balanced upward and downward. In our retrospective study, it can be found that the metastasis rates of the upper mediastinum and the left gastric artery are basically equivalent in the final pathology (data not shown). However, in the long-term follow-up, a high rate of recurrence was found in the upper mediastinum but rarely in the lower mediastinum and abdominal cavity. The results indicate that lymph node recurrence may occur in both upstream and downstream after ESD in early-stage patients. Therefore, the design of radiation target of should cover the longer longitudinal and abdominal cavity, not just the lesion.

However, there are two main limitations of this trial. First, owing to the different interventions, the study is not double-blinded. And the recruitment may be challenging because of the different interventions and cost of treatments. Second, the sample size calculation was based on our clinical observation and the previous report.

## Trial status

The trial began recruitment in November 2019. The anticipated time of study completion will be December 2027 if necessary. The protocol version 2.0 was discussed on October 25, 2019, by PIs from all participating centers.

## Data Availability

The datasets used or analyzed (or both) during the present study are available from the corresponding author on reasonable request and in ClinicalTrials.gov, NCT04135664.

## Abbreviations

CRC: Clinical research coordinator
CT: Computed tomography
CTV: Clinical target volume
dCRT: Definitive chemoradiotherapy
DMC: Data monitoring committee
EORTC: European Organization for Research and Treatment of Cancer
ER: Endoscopic resection
ESCC: Esophageal squamous cell carcinoma
ESD: Endoscopic submucosal dissection
GTV: Gross tumor volume
JCOG: Japan Clinical Oncology Group
NCCN: National Comprehensive Cancer Network
OS: Overall survival
PI: Principal investigator
PTV: Planning target volume
QLQ-C30: Quality of Life Questionnaire-Core 30
QLQ-OES18: Quality of Life-Esophageal Module 18 Questionnaire
QoL: Quality of life
RCT: Randomized controlled trial
RFS: Relapse-free survival
TSC: Trial steering committee
